# Long-term outcome and prognostic value of Ki67 after perioperative endocrine therapy in postmenopausal women with hormone-sensitive early breast cancer (POETIC): an open-label, multicentre, parallel-group, randomised, phase 3 trial

**DOI:** 10.1016/S1470-2045(20)30458-7

**Published:** 2020-11

**Authors:** Ian Smith, John Robertson, Lucy Kilburn, Maggie Wilcox, Abigail Evans, Chris Holcombe, Kieran Horgan, Cliona Kirwan, Elizabeth Mallon, Mark Sibbering, Anthony Skene, Raghavan Vidya, Maggie Cheang, Jane Banerji, James Morden, Kally Sidhu, Andrew Dodson, Judith M Bliss, Mitch Dowsett

**Affiliations:** a The Royal Marsden NHS Foundation Trust, London, UK; b The Institute of Cancer Research, London, UK; c University of Nottingham, Nottingham, UK; d Independent Cancer Patients Voice, London, UK; e Poole Hospital NHS Foundation Trust, Poole, UK; f Liverpool University Hospitals Foundation Trust, Liverpool, UK; g Bexley Cancer Centre, Leeds, UK; h University of Manchester and Manchester University NHS Foundation Trust, Manchester, UK; i Queen Elizabeth University Hospital Glasgow, Govan, UK; j University Hospitals of Derby and Burton, Derby, UK; k Royal Bournemouth and Christchurch NHS Foundation Trust, Bournemouth, UK; l University of Birmingham and Royal Wolverhampton NHS Trust, Wolverhampton, UK

## Abstract

**Background:**

Preoperative and perioperative aromatase inhibitor (POAI) therapy has the potential to improve outcomes in women with operable oestrogen receptor-positive primary breast cancer. It has also been suggested that tumour Ki67 values after 2 weeks (Ki67_2W_) of POAI predicts individual patient outcome better than baseline Ki67 (Ki67_B_). The POETIC trial aimed to test these two hypotheses.

**Methods:**

POETIC was an open-label, multicentre, parallel-group, randomised, phase 3 trial (done in 130 UK hospitals) in which postmenopausal women aged at least 50 years with WHO performance status 0–1 and hormone receptor-positive, operable breast cancer were randomly assigned (2:1) to POAI (letrozole 2·5 mg per day orally or anastrozole 1 mg per day orally) for 14 days before and following surgery or no POAI (control). Adjuvant treatment was given as per UK standard local practice. Randomisation was done centrally by computer-generated permuted block method (variable block size of six or nine) and was stratified by hospital. Treatment allocation was not masked. The primary endpoint was time to recurrence. A key second objective explored association between Ki67 (dichotomised at 10%) and disease outcomes. The primary analysis for clinical endpoints was by modified intention to treat (excluding patients who withdrew consent). For Ki67 biomarker association and endpoint analysis, the evaluable population included all randomly assigned patients who had paired Ki67 values available. This study is registered with ClinicalTrials.gov, NCT02338310; the European Clinical Trials database, EudraCT2007-003877-21; and the ISRCTN registry, ISRCTN63882543. Recruitment is complete and long-term follow-up is ongoing.

**Findings:**

Between Oct 13, 2008, and April 16, 2014, 4480 women were recruited and randomly assigned to POAI (n=2976) or control (n=1504). On Feb 6, 2018, median follow-up was 62·9 months (IQR 58·1–74·1). 434 (10%) of 4480 women had a breast cancer recurrence (280 [9%] POAI; 154 [10%] control), hazard ratio 0·92 (95% CI 0·75–1·12); p=0·40 with the proportion free from breast cancer recurrence at 5 years of 91·0% (95% CI 89·9–92·0) for patients in the POAI group and 90·4% (88·7–91·9) in the control group. Within the POAI-treated HER2-negative subpopulation, 5-year recurrence risk in women with low Ki67_B_ and Ki67_2W_ (low–low) was 4·3% (95% CI 2·9–6·3), 8·4% (6·8–10·5) with high Ki67_B_ and low Ki67_2W_ (high–low) and 21·5% (17·1–27·0) with high Ki67_B_ and Ki67_2W_ (high–high). Within the POAI-treated HER2-positive subpopulation, 5-year recurrence risk in the low–low group was 10·1% (95% CI 3·2–31·3), 7·7% (3·4–17·5) in the high–low group, and 15·7% (10·1–24·4) in the high–high group. The most commonly reported grade 3 adverse events were hot flushes (20 [1%] of 2801 patients in the POAI group *vs* six [<1%] of 1400 in the control group) and musculoskeletal pain (29 [1%] *vs* 13 [1%]). No treatment-related deaths were reported.

**Interpretation:**

POAI has not been shown to improve treatment outcome, but can be used without detriment to help select appropriate adjuvant therapy based on tumour Ki67. Most patients with low Ki67_B_ or low POAI-induced Ki67_2W_ do well with adjuvant standard endocrine therapy (giving consideration to clinical–pathological factors), whereas those whose POAI-induced Ki67_2W_ remains high might benefit from further adjuvant treatment or trials of new therapies.

**Funding:**

Cancer Research UK.

**Research in context**

**Evidence before this study**

Longstanding experimental evidence from 1989 led to the hypothesis that short duration, presurgical endocrine therapy for early oestrogen receptor-positive breast cancer might improve clinical outcome. We carried out a PubMed search for relevant clinical studies published from Jan 1, 1989 until Dec 31, 2019 using the terms “neoadjuvant endocrine”, “breast cancer”, “clinical trial”, and “presurgical and endocrine therapy”. No reasonably sized randomised trial addressed this issue by the time POETIC commenced recruitment in 2008. Subsequently, a randomised clinical trial reported that depot progesterone for 5–14 days before surgery improved outcome in node-positive early breast cancer. Before the initiation of POETIC, two small clinical neoadjuvant trials, IMPACT and Z1031, reported that tumour Ki67 2–4 weeks after starting preoperative endocrine treatment predicted outcome better than baseline Ki67. POETIC was designed to establish whether the gain in prognostic accuracy merited routine application of presurgical endocrine therapy for this purpose. An additional PubMed search was done with “Ki67” added to the above search terms. One small study of low dose tamoxifen was identified, but this did not substantially add to the earlier evidence. Another modestly sized trial used to triage patients with 2–4 week Ki67>10% to chemotherapy and reporting the long-term outcome for those less than 10%, has led to a larger ongoing trial. One other large ongoing trial applies 10% as a cutoff at 2 weeks of tamoxifen or an aromatase inhibitor for directing patients to different adjuvant therapy. The concept of complete cell cycle arrest has been developed as an additional possible cutoff for on-treatment Ki67.

**Added value of this study**

Results from POETIC suggest that 2 weeks' preoperative endocrine therapy makes no perceptible improvement in long-term outcome, but was nevertheless a safe treatment practice. The trial confirmed the low risk of recurrence for those with a low baseline Ki67. In patients with a high baseline Ki67 value (>10%) a biopsy 2 weeks after starting preoperative endocrine therapy provides additional clinical utility by predicting long-term outcomes. The trial documents the relationship of 2-week Ki67 with risk of recurrence for estimating whether the prognosis of individual patients is sufficiently good on endocrine therapy alone or whether additional treatment such as chemotherapy or new targeted therapies should be considered.

**Implications of all the available evidence**

The data show no reason for short-term presurgical treatment to be applied for its direct therapeutic potential, but support prescribing an aromatase inhibitor for the short-term period before breast cancer surgery in oestrogen receptor-positive tumours with a high proliferation rate to derive information on early endocrine responsiveness that can be used to predict a patient's 5-year prognosis on standard adjuvant therapy. The clinical manoeuvres to incorporate this in the patient pathway with reliable quality assured Ki67 are straightforward and the measurement of Ki67 is inexpensive, potentially making this an attractive approach to estimating the prognosis of patients with early breast cancer.

## Introduction

The POETIC (Peri-Operative Endocrine Therapy—Individualising Care) trial was designed to address two important hypotheses in the treatment of post-menopausal women with oestrogen receptor-positive early breast cancer.

The first was that short duration presurgical endocrine therapy might improve clinical outcome. This hypothesis was plausible because 2 weeks' preoperative endocrine therapy had been shown to markedly reduce proliferation in human breast cancer as measured by Ki67.^1^, ^2^ Longstanding experimental evidence had shown that the stimulatory effect of surgery on the growth of metastases in mice could be inhibited by perioperative endocrine therapy.^3^, ^4^ Any improvement in long-term outcome following short exposure to preoperative or perioperative endocrine therapy would be achieved with no additional toxicity or resource implications and be of considerable clinical importance.

The second hypothesis concerned identifying which patients with hormone receptor-positive early breast cancer have a sufficiently good prognosis such that standard of care medical treatment, often comprising adjuvant endocrine therapy alone, was sufficient and which group should be considered for additional therapies. Traditional approaches to this problem had used standard prognostic parameters including size, grade, nodal involvement, and age, often integrated into a prognostic tool (eg, Nottingham Prognostic Index,^5^ Adjuvant Online,^6^ NHS PREDICT^7^), but these merely provided the predicted probability of benefit for a patient population with given tumour and demo-graphic characteristics. More recently, genomic platforms have been developed aimed at providing more accurate prognostic and predictive information for the individual patient.^8^, ^9^ However, these genomic tests are expensive, by no means universally available, and differ among themselves in terms of the information they provide.^10^

A simple test which predicts outcome after short duration preoperative endocrine therapy could therefore be helpful in accurately selecting appropriate treatment in the individual patient, if it incorporated an in-vivo response to aromatase inhibitor. A small neoadjuvant trial (IMPACT) had already suggested this might be feasible: results showed that tumour Ki67 after 2 weeks (Ki67_2w_) of endocrine treatment predicted outcome better than at baseline (Ki67_B_), remaining significant in multivariable analysis, whereas Ki67_B_ did not.^11^, ^12^ Similar results have subsequently been reported from another small trial comparing letrozole with tamoxifen^13^ and from a further trial comparing anastrozole, letrozole, and exemestane with one another.^14^ POETIC, with a much larger patient population, aimed to build on these findings to provide the definitive clinical evidence to inform future practice.

## Methods

### Study design and participants

This open-label, multicentre, parallel group, randomised, phase 3 trial recruited participants from 130 UK hospitals (appendix p 25–27). Eligible patients were postmenopausal women (aged at least 50 years with amenorrhoea for more than 12 months, bilateral oophorectomy or hysterectomy, or had been on hormone replacement therapy within the previous 12 months, and with follicle-stimulating hormone concentrations in the postmenopausal range if aged less than 55 years) with oestrogen receptor-positive or progesterone receptor- positive (Allred ≥3, H-score ≥2, or ≥1% of positive cells, assessed in local pathology laboratories), HER2-positive or HER2-negative (assessed locally), operable primary breast cancer and no evidence of metastatic spread investigated according to local guidelines. If palpable, a tumour of any size was sufficient, otherwise requiring an ultrasound size of at least 1·5 cm. Women required WHO performance status 0–1 and an indication for standard adjuvant endocrine therapy. Required staging investigations were according to local practice with no additional trial specific investigations. Exclusion criteria were typical for this patient population. Previous endocrine therapy or chemotherapy was not allowed, nor was concurrent use of hormone replacement therapy or any other oestrogen-containing medication (within 4 weeks of randomisation). No previous use of oestrogen implants at any time, current, continuous, long-term systemic steroid usage, or treatment with an unlicensed or investigational drug within 4 weeks of randomisation was allowed. Patients with invasive malignancy diagnosed within the previous 5 years or any severe co-incident medical disease were ineligible (appendix p 1).

Patients provided written informed consent before enrolment. POETIC was sponsored by the Institute of Cancer Research (ICR) and Royal Marsden NHS Foundation Trust and approved by the London–South East Research Ethics Committee (reference 08/H1102/37) and managed and analysed by the ICR Clinical Trials and Statistics Unit (ICR-CTSU; appendix p 1 for study oversight details). The protocol is in the appendix.

### Randomisation and masking

Participants were randomly allocated (2:1) to perioperative aromatase inhibitor (POAI) treatment or no perioperative treatment (control) by computer-generated permuted block method (variable block size six or nine) derived centrally by ICR-CTSU using its dedicated randomisation system, stratified by hospital. To randomly assign a patient, staff at the recruiting site telephoned ICR-CTSU and thus had no knowledge of future treatment assignment. The allocation ratio weighted trial information to study of biological perioperative drug effects, in particular to assess how these effects relate to long-term outcome. No placebo was used; clinicians and patients were not masked to treatment allocation, but central laboratory staff were masked.

### Procedures

POAI was a non-steroidal aromatase inhibitor in standard dosage (oral anastrozole 1 mg per day or oral letrozole 2·5 mg per day); choice of agent was declared by each participating hospital at trial outset. Before randomisation, all patients had excisional surgery prebooked for around 2 weeks (minimum 10 days) later to ensure timing of surgery was not biased by treatment allocation. POAI was to commence immediately after randomisation allowing duration of treatment before surgery to be as close as possible to 14 days. If surgery was delayed, the pretreatment duration was extended. Treatment continued without interruption until 14 days after surgery.

All non-trial adjuvant therapy, laboratory investigations, and disease staging were established on clinical grounds according to standard of care local practice (appendix p 1). All patients had pretreatment mammography and breast ultrasound according to local diagnostic practice. In December, 2010, the independent data monitoring committee expressed caution relating to the potential influence of POAI therapy on tumour grade measured at surgery. In February, 2011, a letter to investigators, followed by an approved protocol amendment, recommended that local multidisciplinary teams gave due consideration to other factors, including pretreatment grade on diagnostic core where available, when considering use of adjuvant chemotherapy.

Follow-up data were submitted annually to ICR-CTSU; disease-related events, second cancers and deaths were reported on occurrence. There was no specific safety endpoint. Adverse event data were restricted to three menopausal symptoms (hot flushes, sweating, and musculoskeletal pain) at baseline, surgery, and at follow-up 2 weeks postsurgery (assessed using National Cancer Institute Common Terminology Criteria for Adverse Events version 3) as the safety profiles of the aromatase inhibitors used were well established. Serious adverse events were reported or recorded (as per protocol). Participants were able to withdraw from the trial at any time for any reason.

Formalin-fixed paraffin-embedded tissue samples were required before randomisation (baseline) and at surgery. Baseline samples could be a core-cut diagnostic biopsy, a subsequent research core-cut biopsy, or sections from the diagnostic sample. At surgery, samples could be either core biopsies or sections cut from the routine excision.

Tissue samples were processed, stored, and analysed for Ki67 staining centrally in the Ralph Lauren Centre for Breast Cancer Research at the Royal Marsden NHS Foundation Trust. Ki67 was analysed immunohistochemically in a core biopsy taken at baseline (Ki67_B_), and in either a core biopsy or the excision biopsy taken at surgery (Ki67_2W_), and was estimated as the percentage of cancer cells staining positive. We used MIB1 as the primary antibody to Ki67 and detection was done with the REAL EnVision system, both from DAKO (Glostrup, Denmark until 2016; now Agilent Technologies, Didcot, UK). Scoring was according to methodology including between-batch quality control procedures as described by the International Ki67 in Breast Cancer Working Group Party.^15^ Analysis of 2-week samples from the control group was restricted to a randomly selected subset since minimal change from baseline was expected.^16^

### Outcomes

The primary endpoint was time to recurrence (time from randomisation to local, regional, or distant tumour recurrence or death from breast cancer without previous notification of relapse) with second primary cancers and intercurrent deaths censored. Secondary clinical endpoints included relapse-free survival (as per time to recurrence but also including deaths from any cause as events), time to local recurrence (time from randomisation to first confirmed local recurrence, censoring at previous distant recurrence, second primary cancer, or death), time to distant recurrence (time from randomisation to first confirmed distant recurrence or breast cancer death without previous relapse, censoring at second primary cancer or intercurrent death) and overall survival (time from randomisation to death from any cause). Breast cancer-free survival duplicated the definition of time to recurrence, and was listed in the protocol in error.

Ki67 was evaluated as a biomarker in relation to its effect on predicting disease outcomes (one of the trial's two key objectives) and as the molecular secondary endpoint to assess proliferation rate at baseline (Ki67_B_) and at surgery (Ki67_2w_), thus assessing the impact of POAI. The additional molecular secondary endpoint of gene expression profile at core biopsy and at surgical excision is not reported here as data analysis is ongoing.

### Statistical analysis

The sample size assumed the proportion of patients with recurrence by 5 years would be low (approximately 10%) given known recurrence rates for similar populations.^17^, ^18^ With 4350 patients it would be possible to detect a 3% improvement in time to recurrence at 5 years (10% to 7% recurrences) with 91% power (two-sided α of 5%). The sample size was increased originally from 4000 to 4350 patients to allow for underestimation of the relapse rate potentially owing to patients dying from other causes before breast cancer relapse. This change was endorsed by the trial steering committee and independent data monitoring committee and managed via a protocol amendment approved on Dec 31, 2012.

Analyses relating to clinical endpoints were done according to modified intention-to-treat—removing patients who subsequently withdrew consent for use of data. For analyses that assessed the predictive value of Ki67_B_ and Ki67_2W_, the population was defined as all randomly assigned patients who had paired Ki67 values available. Patients who did not have primary breast surgery as planned were censored at the date of that decision.

Baseline demographic details, tumour characteristics, adjuvant treatment, and Ki67 data are presented with descriptive statistics. Protocol compliance between treatment groups (time from randomisation to surgery and number of inpatient days for surgery) was compared using Wilcoxon rank-sum tests; differences in tumour grade at surgery were assessed using a χ^2^ test for trend in prespecified analyses. Worst grade of adverse events and serious adverse reactions to POAI were summarised descriptively. Ki67_B_ and Ki67_2W_ were reported by HER2 status. Analysis of percentage change between Ki67_B_ and Ki67_2W_ used Wilcoxon sign rank tests within treatment groups and Wilcoxon rank-sum test between treatment groups. In a post-hoc exploratory analysis, following initial planned analyses on the trial data, a multivariable logistic regression model was created, using a forward stepwise approach, to determine factors affecting chemotherapy use.

For survival-related endpoints, Kaplan-Meier curves were plotted and treatment groups compared with the log-rank test. Hazard ratios (HRs) and 95% CIs were calculated within Cox proportional hazards regression models, with HRs of less than one taken to favour POAI. The proportional hazards assumption was assessed using Schoenfeld residuals and was found to hold. Comparisons between treatment groups were made with and without adjustment for progesterone receptor status (positive, negative, unknown), HER2 status (positive, negative, unknown), presurgical tumour grade (G1, G2, and G3), pathological tumour size (continuous), presurgical histological type (ductal, lobular, special type), nodal status (N0, N1–3, and N4+), age at randomisation (continuous) and vascular invasion (yes, no). Subgroup analyses were done for baseline clinical characteristics and presented using a forest plot.

Associations between Ki67_B_ and Ki67_2W_ and time to recurrence were done separately in the POAI and control groups with the principal focus being to study the on-treatment effect of POAI. A post-hoc analysis of all patients combined for Ki67_B_ was included for completeness. Assessment of Ki67 in the control group was considered of low additional value because patients were not exposed to perioperative treatment and because of the lack of association between POAI and time to recurrence. Survival analysis included adjustment for clinical factors as mentioned previously, except for HER2 status which was a stratifying factor. HER2-positive tumours have a different pattern of recurrence and were typically additionally treated with specific HER2-targeted therapy. To explore associations between Ki67 and disease outcome in the POAI group, Ki67 scores were dichotomised and patients divided into four groups as follows: low–low (Ki67_B_ and Ki67_2W_ <10%); high–low (Ki67_B_ ≥10%, Ki67_2W_ <10%); high–high (Ki67_B_ and Ki67_2W_ ≥10%); and low–high (Ki67_B_ <10%, Ki67_2W_ ≥10%). Few POAI patients were classified into the low–high group. These are reported for completeness but not further analysed as their apparent response is probably due to measurement variability around the dichotomisation cut-point. Post-hoc subgroup analyses explored associations between Ki67 and disease outcome by chemotherapy use and age with a view to avoid confounding of interpretation. In addition to the predefined 10% Ki67 dichotomisation, chosen to ensure consistency with other neoadjuvant trials,^12^, ^14^ other cut-points were explored using Harrell's C coefficient^19^ including that for complete cell cycle arrest (CCCA; Ki67 ≤2·7%^20^).

Previous analyses^21^ of change in Ki67 in 679 control group patients with paired samples available indicated that in patients with a core-cut surgery sample the median proportional reduction was −4·1% (IQR −27·8 to 34·8), whereas in those with a resection sample at surgery, the median proportional reduction in Ki67 between baseline and surgery was −17·7% (IQR −44·2 to 12·7) in contrast with an earlier small pilot study.^16^ From these findings, it was assumed that, for a given surgical sample, change in Ki67 score would be proportionally approximately 15% less if the sample was core-cut rather than resection (eg, 10% reduction with resection sample translated to 8·5% for core-cut). To account for this difference, Ki67 data and the analyses linking Ki67 and time to recurrence were done with Ki67_2W_ corrected according to surgical sample type. Ki67_2W_ scores from resection samples were increased proportionally by 15%. This correction factor was derived (and used) in control participants and similarly applied to participants in the POAI group. The correction was also made for patients for whom surgical sample type was unknown. For cases where Ki67_2W_ was 0%, no adjustment was made.

This manuscript describes the primary endpoint analysis, time to recurrence after a 5-year median follow-up for both hypotheses; first by randomised POAI allocation and second exploring the ability of Ki67 to predict disease outcome. No formal interim analyses were planned or done before the primary analysis. For this purpose, a database snapshot was taken on Aug 8, 2017 for data presented at the San Antonio Breast Cancer Conference 2017 and updated with a second database snapshot taken on Feb 6, 2018. All analyses were done by means of Stata (version 13.1). A p value of less than 0·05 was deemed to be significant.

This study is registered with ClinicalTrials.gov, NCT02338310; the European Clinical Trials database, EudraCT2007-003877-21; and the ISRCTN registry, ISRCTN63882543.

### Role of the funding source

The funder of the study had no role in study design, data collection, data analysis, data interpretation, or writing of the report. The corresponding author had full access to all of the data in the study and had final responsibility for the decision to submit for publication.

## Results

**Figure 1:**
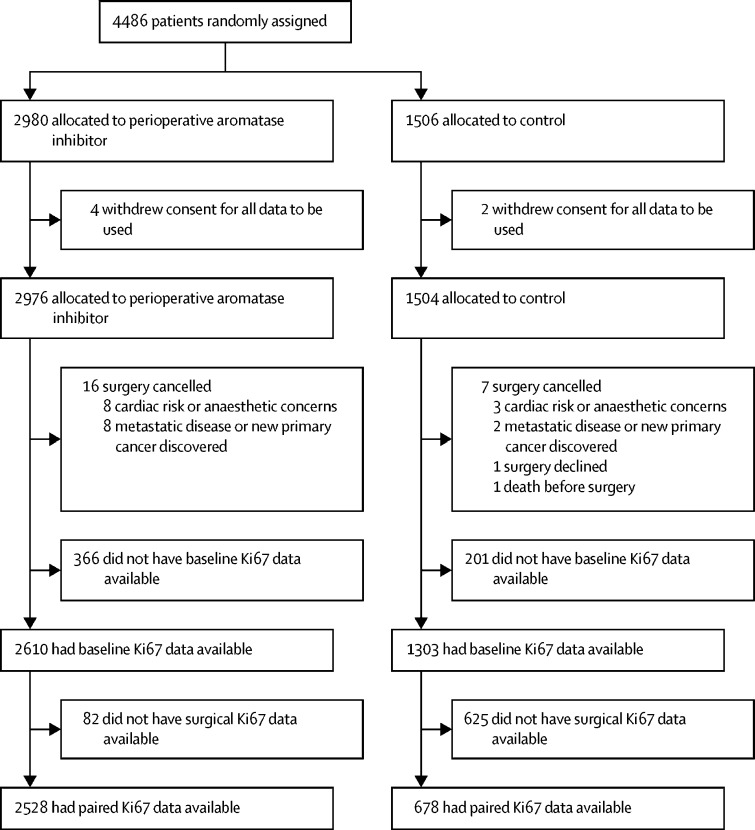
Trial profile

Between Oct 13, 2008, and April 16, 2014, 4486 patients were entered from 130 UK centres. Six patients subsequently withdrew consent for data to be used and therefore 4480 patients (2976 POAI, 1504 control) were included in the modified intention-to-treat analysis (figure 1).

Median age at randomisation was 67·1 years (IQR 61·5–74·8), 2536 (57%) of 4480 patients had a tumour size up to 2 cm, and all but eight (<1%) patients were confirmed locally to have hormone receptor-positive tumours (table 1). 23 (1%) of 4480 patients did not have surgery as planned (16 patients in the POAI group and seven in the control group; figure 1). Adherence to trial treatment and timelines are shown in the appendix (p 14). 177 (6%) of 2976 patients did not have the protocol defined duration of POAI (preoperatively <10 days or >21 days, postoperatively <10 days). The most common reasons were 63 (2%) had their surgery changed, 35 (1%) had less owing to adverse events (16 were in the presurgical period), and 30 (1%) had less owing to patient choice or omission. Surgical details and postsurgery tumour characteristics were well balanced between groups with the exception of pathological tumour grade, which was higher in the control group (p<0·0001; table 1). Adjuvant radiotherapy and anti-HER2 therapy were given after surgery with similar frequency for the two groups and in line with UK standard of care. Adjuvant chemotherapy was given to 770 (26%) of 2957 patients in the POAI group and 460 (31%) of 1493 patients in the control group (appendix p 15) with multivariable analyses attributing this to differences observed in postsurgical grade (appendix p 16). Following surgery, most (POAI 2507 [86%] of 2960 patients; control 1186 [81%] of 1497 patients) women were prescribed aromatase inhibitor monotherapy (appendix p 17).

**Table 1:** Baseline characteristics

			**Demographics at randomisation and tumour characteristics from the diagnostic core**		**Surgery details and tumour characteristics from surgery**	
			Perioperative aromatase inhibitor group (n=2976)	Control group (n=1504)	Perioperative aromatase inhibitor group (n=2960)	Control group (n=1497)
Age group at randomisation, years						
	<50		9 (<1%)	3 (<1%)	..	*..*
	50–59		579 (19%)	291 (19%)	*..*	*..*
	60–69		1245 (42%)	609 (40%)	*..*	*..*
	70–79		808 (27%)	429 (29%)	*..*	*..*
	≥80		335 (11%)	172 (11%)	*..*	*..*
Age, years			67·1 (61·5–74·9)	67·3 (61·5–74·8)	*..*	*..*
Planned aromatase inhibitor						
	Anastrozole		954 (32%)	483 (32%)	*..*	*..*
	Letrozole		2022 (68%)	1021 (68%)	*..*	*..*
Hormone receptor status						
	Positive		2971 (100%)	1501 (100%)	*..*	*..*
	Negative		4 (<1%)	1 (<1%)	*..*	*..*
	Missing		1 (<1%)	2 (<1%)	*..*	*..*
HER2 status						
	Positive		*..*	*..*	317 (11%)	152 (10%)
	Negative		*..*	*..*	2606 (88%)	1316 (88%)
	Unknown or missing		*..*	*..*	37 (1%)	29 (2%)
	Hormone receptor and HER2 status*					
	Hormone receptor-positive					
		HER2 positive	*..·*	*..*	317 (11%)	152 (10%)
		HER2 negative	*..*	*..*	2606 (88%)	1316 (88%)
		HER2 unknown	*..*	*..*	37 (1%)	29 (2%)
	Hormone receptor-negative					
		HER2 negative	*..*	*..*	4 (<1%)	1 (<1%)
Histological type						
	Ductal†		2404 (81%)	1198 (80%)	2364 (80%)	1199 (80%)
	Lobular		428 (14%)	224 (15%)	454 (15%)	236 (16%)
	Special type‡§		105 (4%)	58 (4%)	124 (4%)	50 (3%)
	Ductal carcinoma in situ or lobular carcinoma in situ¶		0	0	3 (<1%)	2 (<1%)
	Not breast cancer‖		0	0	1 (<1%)	0
	Not known		39 (1%)	24 (2%)	14 (<1%)	10 (1%)
Tumour grade						
	G1		417 (14%)	234 (16%)	465 (16%)	184 (12%)
	G2		1757 (59%)	843 (56%)	1968 (66%)	838 (56%)
	G3		521 (18%)	279 (19%)	502 (17%)	463 (31%)
	GX		0	1 (<1%)	..	..
	Not known**		278 (9%)	145 (10%)	17 (1%)	6 (<1%)
	Missing		3 (<1%)	2 (<1%)	8 (<1%)	6 (<1%)
Tumour size, cm††						
	≤2		1666 (56%)	870 (60%)	1372 (46%)	671 (45%)
	>2–5		1238 (42%)	599 (40%)	1448 (49%)	745 (50%)
	>5		54 (2%)	28 (2%)	129 (4%)	74 (5%)
	Missing		18 (1%)	7 (<1%)	11 (<1%)	7 (<1%)
Definitive breast surgery						
	Mastectomy		..	..	1051 (36%)	503 (34%)
	Conservative surgery		..	..	1902 (64%)	992 (66%)
	Missing		..	..	7 (<1%)	2 (<1%)
Definitive axillary surgery						
	Yes		..	..	2911 (98%)	1470 (98%)
	Clearance		..	..	916 (31%)	468 (31%)
	Sampling		..	..	287 (10%)	150 (10%)
	Sentinal lymph node biopsy		..	..	1708 (58%)	852 (57%)
	No		..	..	42 (1%)	25 (2%)
	Missing		..	..	7 (<1%)	2 (<1%)
Nodal status						
	N0		..	..	1815 (61%)	892 (60%)
	N1–3		..	..	801 (27%)	434 (29%)
	N4+		..	..	334 (11%)	165 (11%)
	Missing		..	..	10 (<1%)	6 (<1%)
Vascular invasion						
	Yes		..	..	813 (27%)	445 (30%)
	No		..	..	1990 (67%)	981 (66%)
	Not reported		..	..	143 (5%)	63 (4%)
	Missing		*..*	*..*	14 (<1%)	8 (1%)
Multi-focal disease						
	Yes		*..*	*..*	381 (13%)	223 (15%)
	No		*..*	*..*	2563 (87%)	1266 (85%)
	Missing		*..*	*..*	16 (1%)	8 (1%)

Data are n (%) and median (IQR). Surgery details exclude patients for whom surgery was permanently cancelled.

* One patient (perioperative aromatase inhibitor) with hormone receptor status unknown was HER2 negative; the remaining two patients (control) with hormone receptor status unknown also had HER2 status unknown.

† Ductal includes patients with mixed ductal and lobular tumours.

‡ Special types on the diagnostic core include mucinous, papillary, tubular, metaplastic carcinoma, microcapillary, anaplastic with basaloid nuclear pattern.

§ Special types from surgery specimen include mucinous, papillary, tubular, endocrine cell carcinoma, pure special type, metaplastic carcinoma clear cell, and basaloid, tubular, and cribiform carcinoma.

¶ Presurgical histological types for these patients were coded as ductal carcinoma.

‖ Prehistological type was not known (this patient is recorded as ineligible).

** Some UK hospitals do not routinely report grade on the diagnostic core.

†† Presurgery this measurement is either by ultrasound or clinical examination. Patients are eligible if they have either a palpable tumour (clinical examination) of any size or a tumour with an ultrasound size of ≥1·5 cm. 618 patients had tumour size <1·5 cm, of which 607 had a tumour confirmed as palpable.

**Figure 2:**
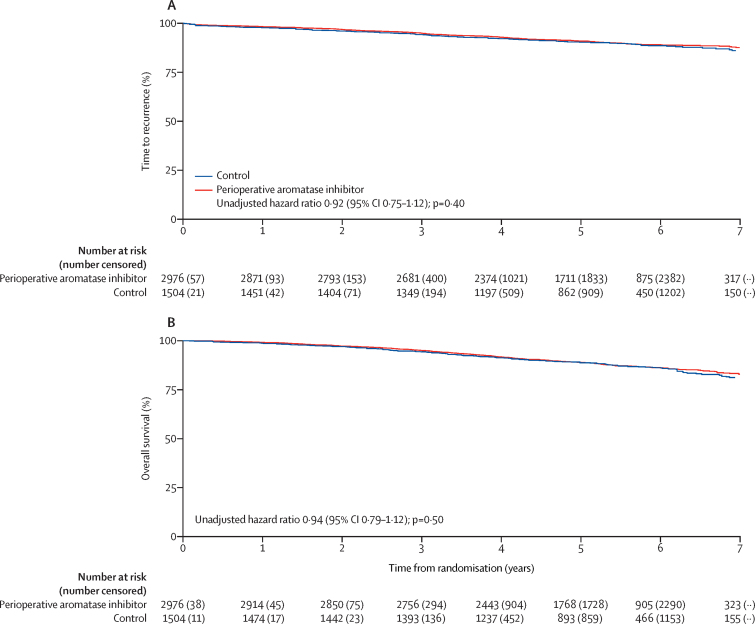
Kaplan-Meier survival curve by randomised treatment group for time to recurrence (A) and overall survival (B) In part A test for proportionality, p=0·58. In part B test for proportionality, p=0·82.

With 62·9 months' median follow-up (IQR 58·1–74·1), 434 (10%) of 4480 women had a breast cancer recurrence (POAI 280 [9%] of 2976 patients, control 154 [10%] of 1504 patients; table 2) with no significant difference observed between the treatment groups (HR 0·92, 95% CI 0·75–1·12; p=0·40, adjusted HR 0·96, 0·77–1·19; p=0·70; figure 2A) with the proportion free from breast cancer recurrence at 5 years of 91·0% (89·9–92·0) in the POAI group and 90·4% (88·7–91·9) in the control group. Subgroup analyses according to clinical characteristics, including nodal status, were consistent with the overall effect (appendix p 2). Likewise, no significant differences between treatment groups were observed for relapse-free survival, time to local recurrence, and time to distant recurrence (table 3). Second breast cancer primaries developed in 26 (<1%) of 2976 women in the POAI group compared with 24 (2%) of 1504 in the control group. 561 patients had died (POAI 365 [12%] of 2976; control 196 [13%] of 1504). Almost half of deaths were attributable to a non-breast cancer cause; none were treatment related (table 2). There was no difference in overall survival between treatment groups. 5-year overall survival was 88·9% (95% CI 87·7–90·1) in the POAI group versus 88·9% (87·2–90·5) in the control group (unadjusted HR 0·94, 95% CI 0·79–1·12; p=0·50, adjusted HR 0·91, 0·75–1·10; p=0·33; figure 2B).

**Table 2:** Disease-related first events and deaths

	**Perioperative aromatase inhibitor group (n=2976)**	**Control group (n=1504)**
**Any disease-related first event**		
Yes	541 (18%)	309 (21%)
No	2435 (82%)	1195 (80%)
**Event contributing to primary endpoint (time to recurrence)**		
Total	280 (9%)	154 (10%)
Local recurrence (isolated)	25 (1%)	13 (1%)
Distant recurrence*	236 (8%)	131 (9%)
Breast cancer death†	19 (1%)	10 (1%)
**Other event**		
Total	261 (9%)	155 (10%)
Breast second primary cancer	26 (1%)	24 (2%)
Non-breast second primary cancer	136 (5%)	80 (5%)
Intercurrent death	99 (3%)	51 (3%)
**Deaths**		
Total	365 (12%)	196 (13%)
Breast cancer	201 (7%)	110 (7%)
Other (intercurrent deaths)	164 (6%)	86 (6%)
Cardiovascular	41 (1%)	25 (2%)
Other cancer	59 (2%)	35 (2%)
Respiratory	37 (1%)	15 (1%)
Sepsis	14 (<1%)	5 (<1%)
Other‡	13 (<1%)	6 (<1%)

Data are n (%). If more than one first event was reported on the same date, it was included in the row here according to the following order of priority: distant recurrence, local recurrence, breast second primary cancer, non-breast second primary cancer, and intercurrent death.

* Distant recurrence row included patients for whom distant recurrence is reported within 6 weeks of local recurrence.

† Included 25 patients (18 perioperative aromatase inhibitor, seven in the control group) with unknown cause of death and no previous event; one patient had a second primary cancer before unknown cause of death and was not included here.

‡ Other causes in the perioperative aromatase inhibitor group (n=13) were accident (n=2), acute kidney injury, Alzheimer's disease, ascending aortic aneurysm, haematemesis secondary to gastric ulcer, hepatic cirrhosis, multiorgan failure, myelofibrosis, old age with vascular deterioration and chronic kidney disease, portal hypertension, a fall, ascites, evidence of cirrhosis, postoperative complications relating to pituitary tumour operation, and renal failure; other causes in the control group (n=6) were complications post laparotomy, dementia, diabetes, meningioma, subdural haematoma, and suicide.

**Table 3:** Summary of disease-related endpoints

	**Number of events**		**Unadjusted hazard ratio**	**Adjusted hazard ratio**	**5-year survival estimate**	
	Perioperative aromatase inhibitor group	Control group			Perioperative aromatase inhibitor group	Control group
Relapse-free survival	385 (13%)	207 (14%)	0·94 (0·79–1·11); 0·47	0·95 (0·79–1·14); 0·59	87·9% (86·6–89·1)	87·6% (85·7–89·2)
Time to local recurrence	41 (1%)	24 (2%)	0·86 (0·52–1·43); 0·57	0·92 (0·54–1·56); 0·75	98·6% (98·1–99·0)	98·5% (97·6–99·0)
Time to distant recurrence	262 (9%)	147 (10%)	0·90 (0·73–1·10); 0·30	0·94 (0·75–1·18); 0·59	91·7% (90·5–92·6)	90·9% (89·2–92·3)

Data are n (%), hazard ratio (95% CI); p value, and % (95% CI). Models adjusted for progesterone receptor status (positive, negative, unknown), HER2 status (positive, negative, unknown), presurgical tumour grade (G1, G2, and G3), pathological tumour size (continuous), presurgical histological type (ductal, lobular, special type), nodal status (N0, N1–3, and N4+), age at randomisation (continuous), and vascular invasion (yes, no). Test for proportionality for relapse-free survival, p=0·69; for time to local recurrence, p=0·97, and for time to distant recurrence, p=0·52.

Selected menopausal symptoms were assessed in 4201 (94%) of 4480 women, with higher symptom rates observed for POAI (appendix p 18). The most commonly reported grade 3 adverse events were hot flushes (20 [1%] of 2801 patients in the POAI group *vs* six [<1%] of 1400 in the control group) and musculoskeletal pain (29 [1%] *vs* 13 [1%]). 11 patients each reported a single serious adverse reaction (appendix p 19); all in the POAI group. The most common were pulmonary embolism (n=3) and musculoskeletal pain (n=3).

3913 (87%) of 4480 participants had Ki67_B_ data available. 2528 (85%) of 2976 patients in the POAI group and 678 (45%) of 1504 in the control group had paired Ki67_B_ and Ki67_2W_ data available (figure 1). In 2316 (72%) of 3206 participants with paired Ki67 data, the surgical sample was a resection (1834 [73%] of 2528 patients in the POAI group; 474 [70%] of 678 patients in the control group) or the surgical sample type was unknown (six in the POAI group and two in the control group) and the Ki67_2W_ scores for these resections and unknown surgical sample types were corrected as described. 688 (27%) of 2528 POAI and 202 (30%) of 678 control patients' surgical sample type was core-cut biopsy.

The median Ki67_B_ score in the 3913 of 4480 patients with a sample available was 15·2% (IQR 8·6–26·0; POAI 15·3% [8·5–26·4]; control: 14·9% [8·6–25·1]). Ki67_B_ values were different between HER2-negative and HER2-positive tumours (median 14·3% [IQR 8·2–24·6] in HER2-negative tumours, median 26·6% [17·0–37·4] in HER2-positive tumours; p<0·0001). After 2 weeks of POAI, Ki67 was significantly suppressed compared with little change in the control group. Ki67_2W_ was markedly lower in the HER2-negative tumours compared with HER2-positive tumours (appendix p 3). In the control group, given the little overall change, Ki67_2W_ was again lower in the HER2-negative tumours than in HER2-positive tumours (appendix p 3).

**Figure 3:**
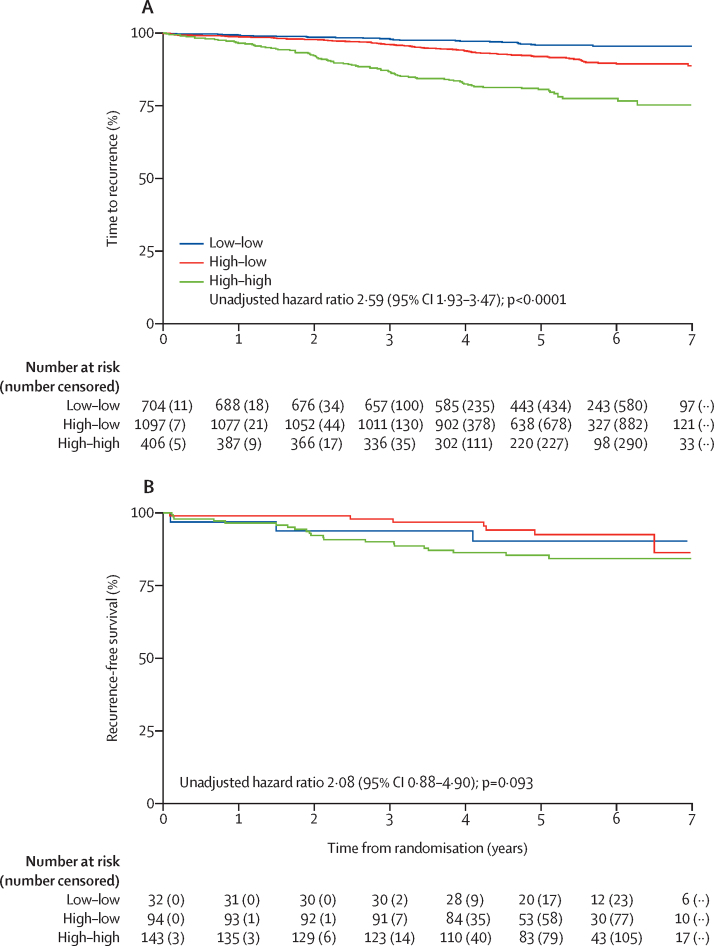
Kaplan-Meier survival curve for time to recurrence by Ki67_B_ and Ki67_2W_ for patients with hormone receptor-positive and HER2-negative breast cancer (A) and hormone receptor-positive, HER2-positive breast cancer (B) in the perioperative aromatase inhibitor group Low–low=Ki67_B_ and Ki67_2W_ <10%. High–low=Ki67_B_ ≥10% and Ki67_2W_ <10%. High–high=Ki67_B_ and Ki67_2W_ ≥10%. B=baseline. 2w=2weeks. 28 patients with hormone receptor-positive breast cancer and HER2-negative breast cancer and four patients with hormone receptor-positive and HER2-positive breast cancer in the low-high group were omitted from the figure.

In patients with HER2-negative tumours in the POAI group (2235 of 2528 patients), 209 (9%) time to recurrence events were reported. For the time to recurrence endpoint, women with Ki67_B_ less than 10% (732 [33%] of 2235 patients) had a better prognosis than those with a Ki67_B_ of at least 10% (1503 [67%] of 2235 patients; appendix p 20). Women whose Ki67_2W_ remained high (high–high group) were significantly more likely to have a recurrence than those whose Ki67_2W_ had dropped below 10% (high–low group; unadjusted HR 2·59, 95% CI 1·93–3·47; p<0·0001, adjusted HR 2·10, 1·48–2·98; p<0·0001; figure 3A). Within the POAI-treated HER2-negative subpopulation, 5-year recurrence risk in women with low Ki67_B_ and Ki67_2W_ (low–low) was 4·3% (95% CI 2·9–6·3), 8·4% (6·8–10·5) with high Ki67_B_ and low Ki67_2W_ (high–low) and 21·5% (17·1–27·0) with high Ki67_B_ and Ki67_2W_ (high–high). Within the POAI-treated HER2-positive subpopulation, 5-year recurrence risk in the low–low group was 10·1% (95% CI 3·2–31·3), 7·7% (3·4–17·5) in the high-low group, and 15·7% (10·1–24·4) in the high–high group. Adding a high versus low classification at 2 weeks segregated groups in relation to their baseline Ki67 (appendix p 21).

The HER2-negative POAI-treated subpopulation post-hoc exploratory analyses relating to the combined effects of age and chemotherapy use suggested that in patients with Ki67_B_ of at least 10%, who did not receive adjuvant chemotherapy, the residual Ki67_2W_ (high or low) conferred a differential effect on prognosis as assessed by time to recurrence for both those aged less than 70 years and aged at least 70 years (appendix pp 4–9). Numbers were too small to fully define effects for the corresponding group (ie, Ki67_B_ ≥10%) who did receive chemotherapy.

In patients with HER2-negative breast cancer in the control group, 56 time to recurrence events were reported in the 597 of 678 patients for whom Ki67_2W_ was available. There was no difference in time to recurrence between the high–high and high–low groups (appendix pp 10, 22).

A post-hoc sensitivity analysis in the HER2-negative subgroup combining the baseline data for POAI and control gave a 5-year recurrence risk of 4·7% (95% CI 3·5–6·3) for low Ki67B and 11·5% (95% CI 10·1–13·1) for high Ki67B (appendix p 24).

Prespecified exploratory analysis in the HER2-negative subgroup suggested an optimal cut-point around 15–20% for Ki67_B_ and around 6–8% for Ki67_2W_ and that using the CCCA threshold for Ki67_2W_ had prognostic discrimination (appendix pp 11–13).

In patients with hormone receptor-positive, HER2-positive breast cancer in the POAI group (273 [10%] of 2528 patients), 33 time to recurrence events were reported. 143 women in the Ki67 high–high group had a recurrence compared with 94 in the high–low group, although the difference was not significant (unadjusted HR 2·08, 95% CI 0·88–4·90; p=0·093, adjusted HR 1·83, 0·71–4·73; p=0·21; figure 3B). Similar to the HER2-negative group, absolute risk of recurrence at 1, 3, and 5 years was higher in the high-high group than in the high–low group (appendix p 23). 5-year recurrence risk in the low-low group was 10·1% (95% CI 3·2–31·3), 7·7% (3·4–17·5) in the high–low group, and 15·7% (10·1–24·4) in the high–high group. In the 70 women with HER2-positive breast cancer in the control group, nine time to recurrence events were reported.

## Discussion

POETIC is, to our knowledge, the largest trial of its kind to assess the potential of POAI therapy in patients with postmenopausal, hormone receptor-positive early breast cancer and it did not show any significant long-term improvement in disease outcomes with this approach. This was despite preclinical experimental evidence in a mouse model suggesting the contrary.^3^, ^4^ A smaller phase 3 clinical trial, which reported after POETIC was initiated, randomly assigned operable breast cancer patients (n=976, 50% hormone receptor-positive, 45% hormone receptor-negative, and 5% hormone receptor unknown) to surgery or an intramuscular injection of depot hydroxyprogesterone 500 mg 5–14 days before surgery; no significant benefit was observed in the overall population (HR 0·87, 95% CI 0·68–1·09; p=0·23), but the results suggested a hypothesis-generating potential disease-free survival improvement in node-positive subgroups (HR 0·72, 0·54–0·97; p=0·02).^22^ In contrast, consistent with the overall finding, POETIC showed no suggestion of long-term outcome improvement with POAI overall or in the node-positive subgroup.

In POETIC, the frequency of chemotherapy was slightly lower in patients in the POAI group than in those in the control group. Multivariable regression supported the suggestion that this was probably because of multidisciplinary teams being influenced by pathological tumour grade, which was on average lower in the patients in the POAI group. This absolute difference was small however (5%), and since the overall event rate was less than 20% would have had an imperceptible effect on outcome comparisons.

On a pragmatic note, it is common practice to start some patients on preoperative endocrine therapy if there has to be a significant delay in surgery for any reason. Despite not showing any statistical evidence of clinical benefit, our results provide reassurance that there is no detriment to this practice.

The second aim of this trial was to explore whether the measurement of tumour Ki67 2 weeks after starting treatment could predict disease outcome better than baseline Ki67 alone, thus providing the basis of a simple and inexpensive test to personalise adjuvant treatment in patients with hormone receptor-positive, HER2-negative breast cancer. Previously, IMPACT had shown that 2-week on-treatment Ki67 predicted outcome better than baseline and, unlike baseline, was significant in multivariable analysis.^12^ POETIC has provided evidence for the clinical validity of on-treatment aromatase inhibitor Ki67_2W_ in addition to Ki67_B_ to predict those with high residual risk of recurrence in spite of standard-of-care therapy. At the initiation of POETIC, we believed that the evidence was insufficient to withhold or direct therapy on the basis of the Ki67_2W_. Our results provide an early indication of endocrine sensitivity or resistance including for the large number of patients who are not routinely considered for adjuvant chemotherapy.

Separate clearly defined adjuvant treatment pathways for HER2-positive and HER2-negative breast cancers now exist and we therefore analysed these groups separately when considering prognostic risk. The HER2-positive subgroup was small with relatively few events. Focus for exploratory analysis was therefore on the HER2-negative subgroup, which comprised approximately 90% of the POETIC population.

Previously, it had been shown that patients with a low Ki67_B_ have a better prognosis than those with a high Ki67_B_ value.^23^ POETIC confirmed this in a larger prospective population, dichotomising Ki67_B_ at 10% with 5-year recurrence risk in HER2-negative patients in the POAI group of 4·4% for low Ki67_B_ and 11·8% for high Ki67_B_. To our knowledge, this is the first large published dataset that makes use of the Ki67 scoring methodology recommended by the International Ki67 in Breast Cancer Working Group; the strong association of Ki67 at baseline with prognosis served as a clinical validation of that methodology.^15^ Patients whose Ki67_B_ was low did well on standard of care, with approximately 85% of those receiving endocrine therapy alone. It could be the case that if the patient's clinical pathological features led to chemotherapy being given, this approach might have contributed to the good outcomes. But irrespective of adjuvant treatment, it is reasonable to conclude that Ki67_2w_ did not add significant prognostic or predictive information in this subgroup.

In contrast, for patients whose tumours had a high baseline Ki67 in the POAI group, 73% had a low Ki67_2w_ 2 weeks after starting treatment; those patients had a better prognosis at 5 years than those who continued to have a high Ki67_2W_ (8·4% *vs* 21·5% 5-year recurrence risk). To what extent could this observation be applied to clinical practice?

The answer to this question is influenced by the limitations of this trial. The first concerns the optimal cutoff for Ki67, and we have shown that dichotomising for cutoffs other than 10% merit further exploration. The second limitation concerns interpreting the data in relation to age and chemotherapy usage. Older age has already been shown to be an independent prognostic factor in breast cancer^24^ and POETIC patients aged at least 70 years had poorer outcomes than those aged below 70 years. Since a substantial minority (26%) of POAI patients had adjuvant chemotherapy, this could be a potential confounding factor in the interpretation of Ki67_2W_ in relation to prognosis and prediction of the value of endocrine therapy alone. To address this, we repeated our analyses in patients according to their receipt of adjuvant chemotherapy. This confirmed a persisting worse outcome for tumours high–high after 2 weeks of an aromatase inhibitor compared with high–low in the 74% of patients not receiving chemotherapy. In the corresponding groups who received chemotherapy, numbers were insufficient to determine a prognostic Ki67 effect or to define a plausible beneficial chemotherapy effect.

In the two-thirds of patients below the age of 70 years not receiving chemotherapy, the overall outcome in terms of recurrence risk was better, probably reflecting the choice of omitting chemotherapy for better prognosis patients. But the key point was that in this population of patients non-confounded by chemotherapy, 21% with high Ki67_B_ remained high at surgery (high–high) and those had 11·2% 5-year recurrence risk (arguably meriting chemotherapy in addition), compared with the low–low groups in which recurrence by 5 years was only 1·6% and the high–low group in which recurrence by 5 years was only 2·9% (indicating that additional chemotherapy would be of no clinically relevant benefit). This exploratory outcome must be interpreted with caution but further supports the prognostic value of measuring Ki67 at 2 weeks.

Similar findings were observed for patients aged at least 70 years. Only 59 of those patients received chemotherapy, too few to provide statistical confidence in the relationship between Ki67 and outcome. In those aged at least 70 years who did not receive chemotherapy, there was again a large difference in outcome between the high–low and high–high groups (5-year recurrence risk 12·3% *vs* 34·5%), again supporting the discriminatory power of measuring Ki67 at 2 weeks, even though the absolute risks were greater.

The prespecified Ki67_2w_ 10% cut-point was chosen for consistency with ongoing clinical trials (ALTERNATE [NCT01953588]; ADAPT [NCT01779206]). The relationship of Ki67_2w_ with recurrence risk is continuous and as illustrated by our analysis by means of CCCA, other cut-points might be selected if appropriate for a specific use (eg, assessing the value of well-tolerated additional treatment).

In conclusion, in POETIC, giving perioperative endocrine therapy with an aromatase inhibitor had no significant effect on long-term outcome. The trial also showed that using Ki67_B_ and aromatase inhibitor on-treatment Ki67_2w_ could help guide adjuvant treatment decisions. First, we believe that we have identified a subgroup with a low baseline Ki67 who have a sufficiently good prognosis that the majority will do well on standard endocrine therapy alone (except perhaps for a minority as dictated by other clinical–pathological factors) and who do not require a repeat 2-week biopsy. Second, giving POAI to the subgroup with high baseline Ki67 can differentiate two groups of patients according to their 2-week Ki67 value: those who convert to a low Ki67 might not need anything beyond adjuvant endocrine therapy (taking consideration of other clinical-pathological factors), whereas those with a high Ki67 that has remained high, should be considered for further adjuvant treatments and trials. There are, of course, now several commercially available genomic platforms developed to provide the same kind of prognostic and predictive information for the individual patient.^8^, ^9^ But these tests are expensive, they often involve central testing of tissue, which has to be sent long distances with inevitable time delay, and results can differ between the platforms. Ki67 as used in POETIC potentially offers an inexpensive, easy and quick alternative in situations in which genomic testing is not readily available.

## Data sharing

De-identified data will be made available to other researchers on request, subject to approval of a formal data access request in accordance with the ICR-CTSU data and sample access policy. Trial documentation including the protocol are available on request by contacting poetic-icrctsu@icr.ac.uk. The ICR-CTSU supports the wider dissemination of information from the research it does, and increased cooperation between investigators. Trial data is collected, managed, stored, shared, and archived according to ICR-CTSU Standard Operating Procedures in order to ensure the enduring quality, integrity, and utility of the data. Formal requests for data sharing are considered in line with the Institute of Cancer Research Clinical Trials and Statistics Unit (ICR-CTSU) procedures with due regard given to funder and sponsor guidelines. Requests are via a standard proforma describing the nature of the proposed research and extent of data requirements. Data recipients are required to enter a formal data sharing agreement which describes the conditions for release and requirements for data transfer, storage, archiving, publication and intellectual property. Requests are reviewed by the Trial Management Group (TMG) in terms of scientific merit and ethical considerations including patient consent. Data sharing is allowed if proposed projects have a sound scientific or patient benefit rationale as agreed by the TMG and approved by the Trial Steering Committee as required. Restrictions relating to patient confidentiality and consent will be limited by aggregating and anonymising identifiable patient data. Additionally all indirect identifiers that might lead to deductive disclosures will be removed in line with Cancer Research UK Data Sharing Guidelines. Additional documents might be shared if approved by the TMG and Trial Steering Committee (eg, statistical analysis plan and informed consent form).
