# Correction to *Lancet Oncol* 2020; 21: 1443–54

**DOI:** 10.1016/S1470-2045(20)30677-X

**Published:** 2020-12

*Smith I, Robertson J, Kilburn L, et al. Long-term outcome and prognostic value of Ki67 after perioperative endocrine therapy in postmenopausal women with hormone-sensitive early breast cancer (POETIC): an open-label, multicentre, parallel-group, randomised, phase 3 trial.* Lancet Oncol *2020; **21:** 1443–54—*In figure 3B of this Article, the y-axis should have read, “Time to recurrence (%)”. This correction has been made to the online version as of Nov 30, 2020.

